# Modulation of the PTPRS proteoglycan switch by antibodies binding to the membrane-proximal fibronectin-type III domain

**DOI:** 10.1016/j.jbc.2025.110470

**Published:** 2025-07-10

**Authors:** Thales Hein Da Rosa, Sterling H. Ramsey, Judy J. Lee, Natalia Y. Kozlova, Zixuan Zhao, Jaeyeon Kim, Ava C. Schneider, Sheng Li, Madhusudhanarao Katiki, Gary S. Firestein, Ramachandran Murali, Eugenio Santelli, Stephanie M. Stanford, Nunzio Bottini

**Affiliations:** 1 Department of Medicine, Kao Autoimmunity Institute, Cedars-Sinai Medical Center, Los Angeles, California, USA; 2 Department of Medicine, University of California, San Diego, La Jolla, California, USA; 3 Department of Biomedical Sciences, Research Division of Immunology, Cedars-Sinai Medical Center, Los Angeles, California, USA; 4 Samuel Oschin Comprehensive Cancer Institute, Cedars-Sinai Medical Center, Los Angeles, California, USA

**Keywords:** tyrosine phosphatase, PTPRS, proteoglycan, antibody, receptor regulation, rheumatoid arthritis, synoviocyte, cell migration

## Abstract

Protein tyrosine phosphatases (PTPs) receptor type II A (R2A) are negatively regulated through oligomerization upon binding of their extracellular domains to glycosaminoglycans (GAGs) on heparan sulfate proteoglycans (HSPGs). Inactivation of receptor PTP sigma (PTPRS) by HSPGs promotes the aggressive behavior of fibroblast-like synoviocytes (FLS) in rheumatoid arthritis (RA). Blocking the binding of its N-terminal, membrane-distal immunoglobulin-like 1 and 2 (Ig1&2) domains to its GAG ligands on the HSPG syndecan-4 (SDC4) promotes PTPRS activity and reverses the pathogenic phenotype of FLS. The potential for therapeutically leveraging other PTPRS ectodomain regions is, however, unknown. We show targeting the membrane-proximal fibronectin type III-like 9 (Fn9) domain offers a novel avenue to activate PTPRS. We mapped PTPRS Fn9 as the binding site of three antibodies (Abs) (13G5, 22H8, 49F2) and characterized their effects on cells. Despite sharing similar epitopes, we found large differences in the ability of these Abs to regulate PTPRS activity. One of these, 13G5, reduced PTPRS-dependent cell migration, PTPRS co-localization with SDC4, and PTPRS oligomerization. Single-chain variable fragment Abs of 13G5 and 22H8 were similarly effective at activating cellular PTPRS as 13G5. Replacing the entire 13G5 constant region enhanced its binding and cellular activity, indicating the Ab's potency can be optimized *via* isotype engineering. Treatment of cells with recombinant Fn9 protein acted as a decoy, disrupting PTPRS colocalization with SDC4 and oligomerization, and inhibiting FLS migration. Finally, significant disease mitigation in mice using 13G5-derived Abs suggests a viable strategy for the generation of novel drugs for RA therapy.

Among post-translational protein modifications, phosphorylation and dephosphorylation of tyrosine residues, catalyzed respectively by protein tyrosine kinases (PTKs) and phosphatases (PTPs), provide a major mechanism for cell signaling owing to rapid modification of protein substrates in response to different stimuli (1, 2). These two classes of enzymes can be exploited to modify cellular behavior for therapeutic purposes (2); however, while several PTK inhibitors are in use in the clinic for indications such as cancer and inflammatory diseases (3), targeting of PTPs lags far behind (4), with the first chemical inhibitors only recently entering clinical trials (5, 6, 7, 8, 9). The human genome encodes over 100 genes for PTPs, including 21 receptor PTPs (RPTP) grouped into eight subtypes (10). Each RPTP is comprised of one or two intracellular catalytic domains, a single transmembrane helix, and a subtype-specific ectodomain (ECD) generally believed to regulate enzymatic activity -a feature that makes RPTPs attractive targets for the development of therapeutically active biologics (11, 12, 13).

Rheumatoid arthritis (RA) is a systemic autoimmune disease characterized by chronic joint inflammation and pain (14). Fibroblast-like synoviocytes (FLS) are joint-resident cells key to maintaining joint structure and function. In RA, FLS acquire a pro-inflammatory phenotype that progressively causes bone and cartilage destruction and are recognized as significant drivers of RA disease (15, 16). The R2A-subtype protein tyrosine phosphatase receptor sigma (PTPRS) is highly expressed in FLS, and we have previously shown that suppression of PTPRS activity strongly contributes to the invasive phenotype typical of FLS in RA (17). R2A PTP activity is regulated by their ∼1250-residue ECD *via* a mechanism coined the proteoglycan (PG) switch, in which binding to GAG chains present on heparan sulfate PGs (HSPGs)—syndecan-4 (SDC4) in the case of PTPRS—leads to oligomerization and inhibition of their enzymatic activity (18, 19, 20). In FLS, PTPRS inhibition results in enhanced migration and invasion through activation of the ezrin-associated cytoskeletal pathway. Function of R2A PTPs can be restored through their detachment from HSPGs, for example, by FLS-expressed PTPRS binding to chondroitin sulfate PGs on the surface of cartilage, leading to reduced FLS migration and invasion (17, 21).

In its major isoform, the PTPRS ECD consists of three N-terminal immunoglobulin (Ig)-like domains, nine fibronectin type III (Fn)-like domains and a ∼15 amino-acid C-terminal linker (Fig. 1*A*) and displays a high degree of internal flexibility (19). Interaction with PGs is mediated by the first two Ig-like domains, denoted as Ig1&2 (18, 22). This knowledge led to the demonstration that soluble versions of PTPRS Ig1&2 acting as a decoy markedly reduce the pathogenic activity of FLS *in vitro* and *in vivo*, providing implications for development of novel RA therapeutics to complement or synergize with existing ones (17, 21). In addition to RA, R2A PTPs are being studied for their roles in other autoimmune diseases through regulation of plasmacytoid dendritic cells (pDC) (23); in neural development, plasticity and regeneration (24); and in cancer as both oncogenes and tumor suppressors (25). Thus, there is a broad interest in gaining a better understanding of how these enzymes are regulated to uncover new strategies for inhibition and enhancement of their activity.

**Figure 1:**
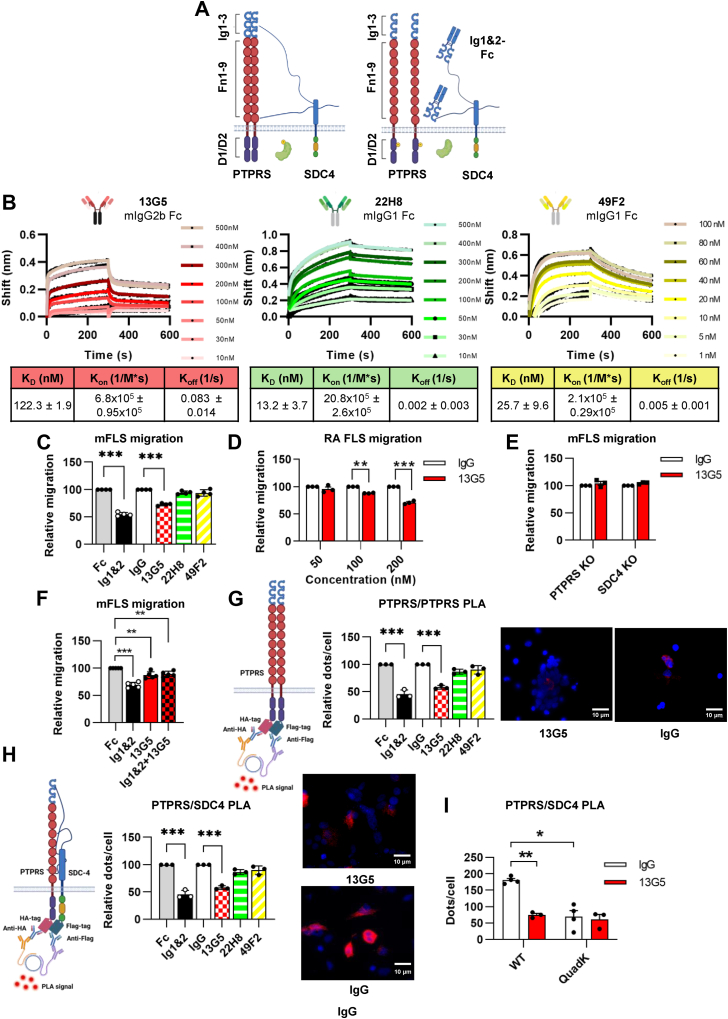
**Anti-PTPRS monoclonal antibody (Ab) 13G5 decreases FLS migration by blocking PTPRS clustering and association with SDC4.***A*, schematic representation of PTPRS and SDC4 interaction with Ig1&2-Fc decoy protein. *B*, 13G5 and 22H8 association/dissociation curves at 500 nM, 400 nM, 300 nM, 200 nM, 100 nM, 50 nM, 30 nM, and 10 nM concentration and 49F2 association/dissociation curves at 100 nM, 80 nM, 60 nM, 40 nM, 20 nM, 10 nM, 5 nM, and 1 nM concentration measured by biolayer interferometry. K_D,_ K_on_ and K_off_ values against PTPRS ectodomain were calculated (n = 3). *C*, relative transwell migration of mouse FLS in the presence of 13G5, 22H8, and 49F2 (200 nM). PTPRS Ig1&2-hFc fusion (Ig1&2) is used for comparison, human Fc and mouse IgG are negative controls. Each dot represents a biological replicate (n = 4). *D*, relative transwell migration of RA FLS in the presence of 13G5 at the indicated concentrations. Mouse IgG is used as a negative control. Each dot represents a biological replicate (n = 3). *E*, relative transwell migration of *Ptprs* and *Sdc4* KO mouse FLS in the presence of 13G5 (200 nM). Each dot represents a biological replicate (n = 3). *F*, relative transwell migration of mouse FLS in the presence of 13G5, Ig1&2, or both 13G5 and Ig1&2 (13G5: 200 nM; Ig1&2: 20 nM). Each dot represents a biological replicate (n = 5). *G*, relative hPTPRS/mPTPRS co-localization by proximity ligation assay (PLA) in the presence of 13G5, 22H8, or 49F2 (200 nM) (*left*) and representative assay images (*right*). Scale bar 10 μm. Ig1&2 is used for comparison. Each dot represents a biological replicate (n = 3). *H*, relative hPTPRS/mSDC4 co-localization by PLA in the presence of 13G5, 22H8, or 49F2 (200 nM) (*left*) and representative assay images (*right*). Scale bar 10 μm. Each dot represents a biological replicate (n = 3). *I*, relative co-localization between hPTPRS WT or QuadK mutant and mSDC4 by PLA in the presence of 13G5 (200 nM). Each dot represents a biological replicate (IgG: n = 4; 13G5: n = 3). Mean ± SEM are shown. ∗∗∗*p* ≤ 0.001, ∗∗*p* ≤ 0.01, and ∗*p* ≤ 0.05 by ordinary one-way ANOVA (*C*–*F*, *H*, and *I*) and Kruskal-Wallis (*G*).

In contrast to the well-characterized ligand-binding function of the Ig1&2 region, little is known about any potential involvement of the remaining Ig-like or Fn-like domains in the molecular mechanism underlying the PG switch. While these could simply function as passive spacers within the protein, it has been hypothesized that self-association and ligand engagement mediated by the Fn5 domain of the R2A PTP LAR is able to modulate its activity (26, 27), suggesting that other R2A PTP domains may play a more active role. Furthermore, it has been shown that the PTPRS membrane-proximal Fn type-III-like domain 9 (Fn9) contributes to the strong *in vitro* affinity of the soluble isolated ECD for heparin, a commonly used HS mimetic, by directly interacting with heparin mainly *via* a string of positively charged amino acids (28). This observation has been linked to the dysregulation of basal PTP activity, although the downstream effect is unclear, has only been demonstrated in the context of a shorter isoform lacking four Fn domains, and its biological relevance has not yet been evaluated (28). Understanding the molecular details of any conformational changes associated with the PG switch would inform the search for novel ways to suppress or promote PTPRS activity. To begin addressing this point, we took advantage of a set of antibodies (Abs) of known sequence directed against the ECD of human PTPRS. We report that Abs recognizing an epitope in the membrane-proximal Fn9 domain inhibit PTPRS oligomerization and FLS migration, phenotypes similarly observed upon release of the PTPRS Ig1&2 region from SDC4 using our previously reported Ig1&2 decoy protein (17, 21). The effectiveness of the anti-PTPRS-Fn9 Ab was dependent on the N-terminal Ig1&2 domains of PTPRS, suggesting distal regulation of the PTPRS PG switch. These data provide new insight into the regulation of R2A PTP intracellular activity through extracellular ligand interactions and suggest new approaches to modulate R2A PTP activity *in vivo*.

## Results

### Anti-PTPRS Ab 13G5 inhibits mFLS migration in a PTPRS and SDC4-dependent manner

We searched the literature for monoclonal Abs (mAbs) against the ECD of PTPRS with publicly available sequences. We focused on a set of 4 mouse Abs, referred to as 9H5 (isotype IgG1/κ), 13G5 (IgG2b/κ), 22H8 (IgG1/κ), and 49F2 (IgG1/κ), developed for recognition of human pDCs (https://patents.google.com/patent/US10730955B2/en) but never, to our knowledge, tested for their effect on FLS migration. We first assessed their ability to recognize the PTPRS ECD using biolayer-interferometry (BLI), measuring kinetics of binding to C-terminally 6xHis tagged mouse PTPRS (mPTPRS) ECD immobilized on an anti-6xHis probe. 22H8 and 49F2 showed the highest affinity for the ECD (K_D_ = 13.2 ± 3.74 and 25.7 ± 9.66 nM, respectively), while the K_D_ for 13G5 was higher (K_D_ = 122 ± 1.9 nM) due to a fast dissociation rate (Fig. 1*B*). 9H5 showed no binding to mPTPRS ECD and was not included in subsequent studies (not shown). When we tested the remaining three Abs at 200 nM concentration in a transwell migration assay using mouse FLS (mFLS), only 13G5 significantly reduced migration, while the other two Abs had only a small, non-statistically significant effect despite their lower *in vitro* K_D_ (Fig. 1*C*). Consistent with the K_D_ value, a dose–response migration assay with 13G5 at 50, 100, and 200 nM showed that lower concentrations were not effective (Fig. S1*A*). To test the efficacy of 13G5 on FLS migration rates in a context relevant to human disease, we performed a dose–response migration assay using human FLS derived from patients with RA (RA FLS) (Fig. 1*D*). 13G5 reduced migration at a concentration of 200 nM and, to a lesser extent, 100 nM. Based on the results with mFLS and RA FLS, we performed subsequent experiments using 13G5 at 200 nM concentration. 13G5 had no effect on *Ptprs* or *Sdc4* KO mFLS migration (Fig. 1*E*), indicating its action on mFLS migration is PTPRS- and SDC4-dependent. We then tested whether 13G5 has an additive or synergistic effect with PTPRS Ig1&2 by performing the migration assay in the presence of Ig1&2, 13G5, or both in combination. Results show treatment with 13G5 + Ig1&2 did not further reduce migration compared to cells treated with Ig1&2 at its maximally effective concentration (Fig. 1*F*), suggesting they act *via* the same cellular mechanism. Finally, the affinity of 13G5 for PTPRS was confirmed using surface plasmon resonance, showing a K_D_ of 95 nM (Fig. S1*B*).

### 13G5 promotes PTPRS activity by blocking its clustering on the surface of cells

According to the current model for inhibition of R2A PTP activity, HSPG binding to the ECD induces clustering of PTP molecules, leading to the organization of its intracellular domains into an inactive, multimeric complex (20). We developed a novel assay to test the ability of Ig1&2 and the three anti-PTPRS Abs to interfere with PTPRS oligomerization and association with SDC4 in cells by proximity ligation assay (PLA). We co-expressed C-terminally Flag-tagged mouse PTPRS (PTPRS-Flag) and HA-tagged human PTPRS (PTPRS-HA) in HEK293T cells and treated them with each of the Abs or Ig1&2. Co-transfection of PTPRS-Flag and PTPRS-HA resulted in a strong PLA signal, showing that PTPRS molecules form a complex in cells (Fig. 1*G*). Treatment of these cells with Ig1&2 or 13G5, but not 22H8 or 49F2, strongly reduced the PLA signal (Fig. 1*G*). These results indicate Ig1&2 and 13G5 reduced PTPRS-PTPRS association and are consistent with their effects on FLS migration. A similar result was obtained in PLA when we examined association between SDC4 and PTPRS: cells co-expressing PTPRS-HA and SDC4-Flag showed strong PLA signal that was significantly reduced by treatment with Ig1&2 or 13G5 but not 22H8 or 49F2 (Fig. 1*H*). 13G5 had no effect on association of SDC4 with a PTPRS mutant unable to bind GAGs (QuadK) (19, 28, 29), likely because the proteins do not appreciably interact already in its absence (Fig. 1*I*), further supporting the notion that the Ab's action on PTPRS is dependent on HSPGs. Taken together, these results suggest that, like Ig1&2, 13G5 blocks SDC4-dependent oligomerization of PTPRS, resulting in activation of PTPRS and reduced FLS migration.

### The anti-PTPRS Abs share an epitope in the PTPRS juxtamembrane region

**Figure 2:**
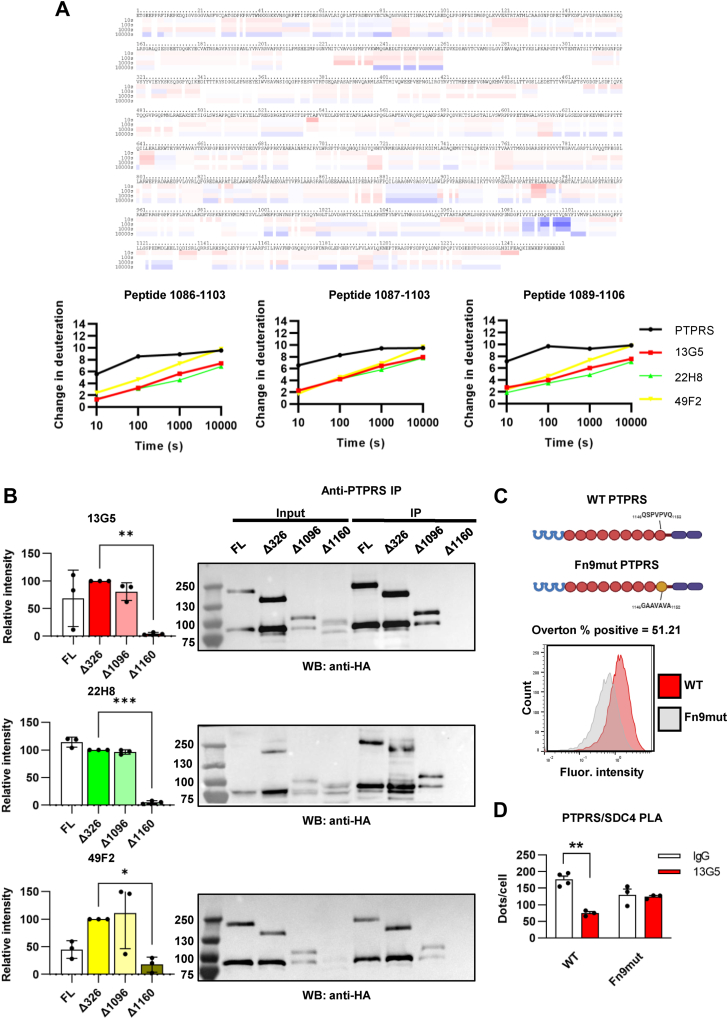
**Anti-PTPRS Abs share an epitope in the PTPRS juxtamembrane region.***A*, hydrogen-deuterium exchange mass spectrometry (HDX-MS) of mouse PTPRS ectodomain in the presence or absence of 13G5, 22H8, and 49F2. Ribbon diagram of the difference between PTPRS alone and PTPRS+13G5 (*top*, blue indicates peptides that exchange faster in the absence of the Ab) and graphs showing the number of hydrogen atoms exchanged at 10, 100, 1000, and 10,000 s for selected peptides (*bottom*). Numbers refer to the sequence of the species used in the experiment, starting at the mature polypeptide. *B*, immunoprecipitation of full-length (FL) or N-terminally truncated PTPRS with 13G5, 22H8, and 49F2 blotted for HA tag. Gel image (*right*) and quantification normalized to PTPRS^Δ^^32^^6^ expression (*left*) are shown. Each dot represents a biological replicate (n = 3). *C*, schematic view of the Fn9 mutation within hPTPRS (*top*). Representative flow cytometry histogram showing 13G5 binding to WT PTPRS and PTPRS Fn9mut (*bottom*). Overton percentage represents the ratio of antibody binding to overexpressed PTPRS. *D*, relative WT or Fn9mut hPTPRS/mSDC4 co-localization in the presence of 13G5 (200 nM). Each dot represents an experimental replicate (WT IgG: n = 4; others: n = 3). Mean ± SEM are shown. ∗∗∗∗*p* ≤ 0.0001, ∗∗∗*p* ≤ 0.001, ∗∗*p* ≤ 0.01, and ∗*p* ≤ 0.05 by ordinary one-way ANOVA (*B*) and Kruskal-Wallis (*D*).

To rationalize the different cellular effects of the three Abs, we next mapped their binding sites on the PTPRS ECD using hydrogen-deuterium exchange mass spectrometry (HDX-MS), in which we measured deuterium exchange rates of amide hydrogens along the polypeptide chain in the absence or presence of an excess of each Ab. Surprisingly, all Abs caused a similar decrease in the fast/medium exchange rate in a region corresponding to a stretch of residues in PTPRS Fn9 (Figs. 2*A* and S2*A*). To validate this result, we used each Ab to immunoprecipitate full-length PTPRS, or three N-terminal truncation mutants respectively lacking Ig-like domains 1 to 3 (PTPRS^Δ326^), the ECD including most of Fn8 (PTPRS^Δ1096^), or the ECD extending into Fn9 past the putative mAb epitope (PTPRS^Δ1160^). As shown in Figure 2*B*, all three Abs precipitated PTPRS-HA that retained the Fn9 domain (full length PTPRS, PTPRS^Δ326^ and PTPRS^Δ1096^) but did not precipitate PTPRS^Δ1160^, suggesting the Fn9 region is required for their binding. To further corroborate these findings, we altered a partially exposed loop (according to the AlphaFold3 pre-computed structural prediction for PTPRS (30)) in the putative Fn9 epitope of hPTPRS by mutating the sequence ^1146^QSPVPVQ^1152^ to GAAVAVA (Fn9mut). We compared binding of 13G5 on the surface of cells transfected with PTPRS or Fn9mut, confirming reduced binding to the latter (Fig. 2*C*). We then performed a PTPRS/SDC4 PLA assay as described in Figure 1, *H* and *I* using HEK293T cells overexpressing either wild-type (WT) or Fn9mut PTPRS along with SDC4 and treated with 13G5 or isotype control. The results in Figure 2*D* show that the mutations in this epitope are sufficient to block the inhibitory effect of 13G5 on PTPRS/SDC4 co-localization. Finally, we confirmed that the three Abs share the same epitope on PTPRS by BLI tandem epitope binning assay. PTPRS ECD was immobilized *via* its C-terminal 6xHis tag and subjected to two consecutive Ab associations. None of the Ab combinations used showed simultaneous binding of the two Abs tested, indicating mutually exclusive binding and suggesting a shared epitope (Fig. S2*B*). Taken together, these findings suggest that the three Abs assessed in this study bind an epitope within the Fn9 domain of PTPRS. Next, we examined the possibility that the lack of an observable effect by some of the antibodies above might be due to their crosslinking of the ECD (an effect previously reported for R2A PTPs (13)), thus compensating for the displacement of GAG binding. As shown in Fig. S2*C*, 22H8 was unable to reverse the effect of Ig1&2 on FLS migration, suggesting that its inability to activate PTPRS is indeed due to a lack of GAG displacement.

### Anti-PTPRS Ab subtype affects its potency *in vitro*

**Figure 3:**
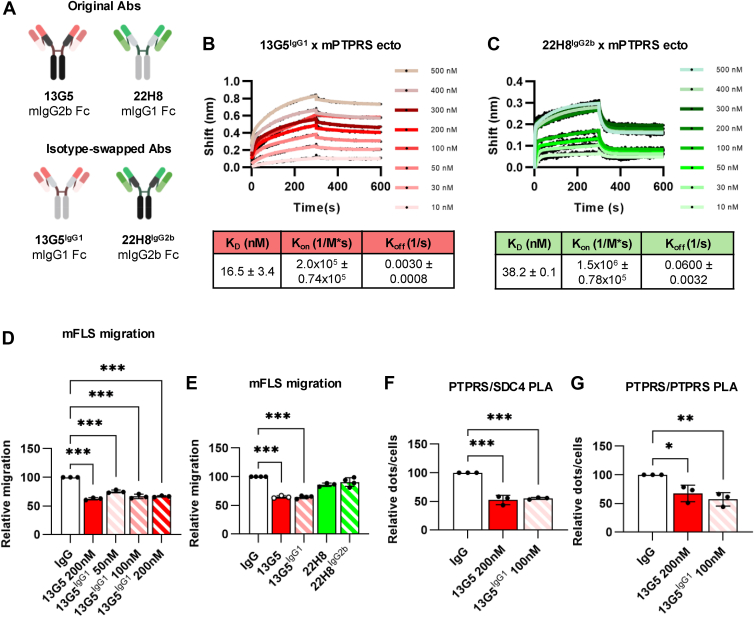
**Ab isotype affects its potency *in vitro*.***A*, schematic representation of 13G5, 22H8, 13G5^IgG1^ and 22H8^IgG2b^ Abs. *B* and *C*, association/dissociation kinetics of 13G5^IgG1^ (*B*) and 22H8^IgG2b^ (*C*) at 500 nM, 400 nM, 300 nM, 200 nM, 100 nM, 50 nM, 30 nM, and 10 nM concentrations measured by bio-layer interferometry (n = 3). *D*, relative transwell migration of mouse FLS in the presence of 13G5 or 13G5^IgG1^ at the indicated concentrations. Each dot represents a biological replicate (n = 3). *E*, relative transwell migration of mouse FLS in the presence of 13G5, 13G5^IgG1^, 22H8 or 22H8^IgG2b^ (200 nM). Each dot represents a biological replicate (13G5 & 22H8: n = 3; others: n = 4). *F*, relative hPTPRS/mSDC4 co-localization by proximity ligation assay (PLA) in the presence of 13G5 (200 nM) or 13G5^IgG1^ (100 nM). Each dot represents a biological replicate (n = 3). *G*, relative hPTPRS/mPTPRS co-localization by PLA in the presence of 13G5 (200 nM) or 13G5^IgG1^ (100 nM). Each dot represents a biological replicate (n = 3). Mean ± SEM are shown. ∗∗∗*p* ≤ 0.001, ∗∗*p* ≤ 0.01, and ∗*p* ≤ 0.05 by ordinary one-way ANOVA (*D–G*).

Having run into an apparent contradiction as the Ab with the lowest *in vitro* affinity for the ECD had the greatest biological activity on both murine and human-derived cells, we first sought to compare *in vitro* affinity as determined by BLI with binding on cells. Both 13G5 and 22H8 bound to HEK293T cells transfected with PTPRS-HA, with 13G5 yielding a ∼2-fold higher signal as assessed by mean fluorescence using a fluorophore-conjugated secondary anti-mouse IgG antibody (Fig. S3). We noticed that the Abs used in this study differ in their subtype, as 13G5 was the only IgG2b/κ subtype mouse Ab, while 22H8, 49F2 and 9H5 were IgG1/κ. To test whether their behavior was dominated by the subtype, we swapped the entire constant region of 13G5 and 22H8, resulting in 13G5^IgG1^ and 22H8^IgG2b^ (Fig. 3*A*) and compared these to the original pair for their *in vitro* affinity to PTPRS and modulation of PTPRS activity in cells. The results show that the specific subtype has a large effect on the affinity and association/dissociation parameters (Fig. 3, *B* and *C*): unlike 13G5, 13G5^IgG1^ has an affinity for PTPRS ECD in the low nanomolar range comparable to that of 22H8, while the K_D_ of 22H8^IgG2b^ is 38.2 nM, again largely driven by a fast dissociation rate. In migration assays, 13G5^IgG1^ displayed near-full activity at 50 nM, suggesting that subtype affects the Ab's potency through its effect on binding affinity but has little, if any, influence on either Ab's broad ability to restore PTPRS activity (Fig. 3*D*). This was confirmed by the continued lack of a significant effect of 22H8^IgG2b^ on FLS migration despite its swapped subtype (Fig. 3*E*) and by PLA demonstrating a marked effect of 100 nM 13G5^IgG1^ at disrupting PTPRS-PTPRS and PTPRS-SDC4 association (Fig. 3, *F* and *G*).

### Single-chain anti-PTPRS Ab fragments decrease mFLS migration, PTPRS/SDC4 proximity, and PTPRS clustering

**Figure 4:**
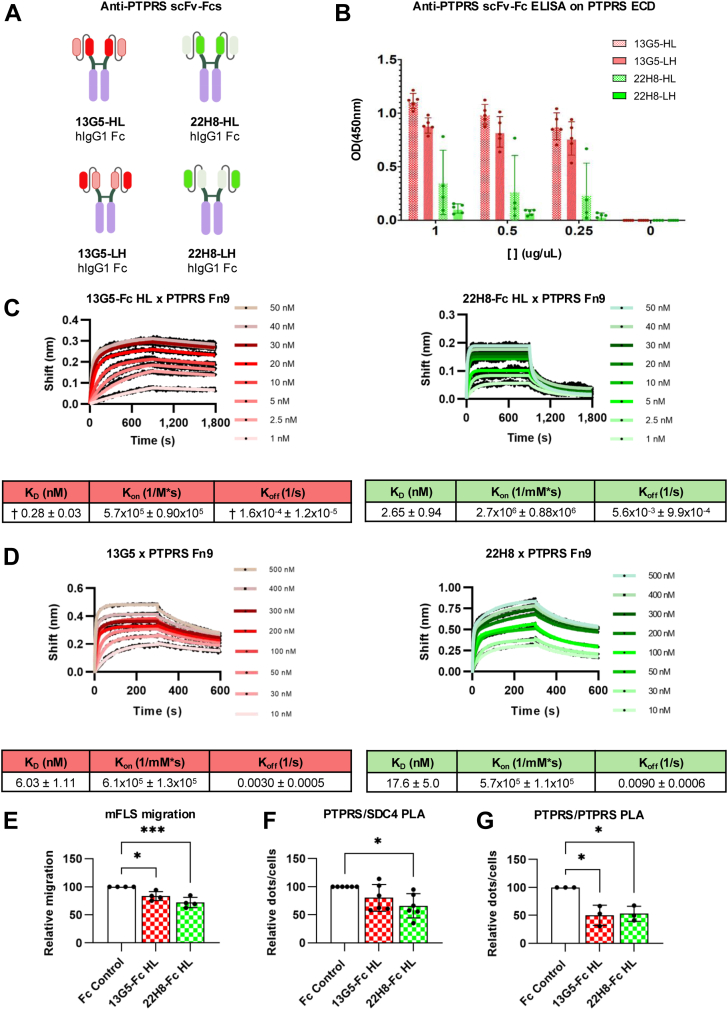
**scFv-Fc fusions reduce FLS migration, PTPRS/SDC4 proximity, and PTPRS clustering.***A*, schematic representation of scFv-Fc fusions. *B*, ELISA showing binding of the scFv-Fcs at different concentrations to the mPTPRS ectodomain. *C*, binding kinetics of scFv-Fc fusion proteins 13G5^HL-scFv-Fc^ (denoted 13G5-HL in the figure) (*left*) and 22H8^HL-scFv-Fc^ (22H8-HL) (*right*) at 50 nM, 40 nM, 30 nM, 20 nM, 10 nM, 5 nM, 2.5 nM, and 1 nM concentrations to immobilized hPTPRS Fn9His by BLI. *D*, binding kinetics of 13G5 and 22H8 at 500 nM, 400 nM, 300 nM, 200 nM, 100 nM, 50 nM, 30 nM, and 10 nM concentration to immobilized hPTPRS Fn9His by BLI. *E*, relative transwell migration of mouse FLS in the presence of scFv-Fc fusion proteins 13G5^HL-scFv-Fc^ (13G5-Fc HL) and 22H8^HL-scFv-Fc^ (22H8-Fc HL) or Fc control protein (200 nM) (n = 4). *F* and *G*, relative hPTPRS/mSDC4 co-localization (*F*) or hPTPRS/mPTPRS clustering (*G*) by proximity ligation assay (PLA) in the presence of scFv-Fc fusion proteins 13G5-Fc HL and 22H8-Fc HL or Fc control protein (200 nM) (*F*: n = 6; *G*: n = 3). Each dot represents a biological replicate. Mean ± SEM are shown. ∗∗∗*p* ≤ 0.001 and ∗*p* ≤ 0.05 by ordinary one-way ANOVA. † indicates that calculated parameters might be inaccurate due to extremely slow dissociation.

We next turned our attention to single-chain variable fragment (scFv) versions of 13G5 and 22F8 in both V_H_-linker-V_L_ and V_L_-linker-V_H_ configurations fused to human IgG1 Fc (scFv-Fc), generating 13G5^HL-scFv-Fc^, 22H8^HL-scFv-Fc^, 13G5^LH-scFv-Fc^, and 22H8^LH-scFv-Fc^ (Fig. 4*A*). Using ELISA, we observed stronger binding for both 13G5 variants to PTPRS ECD, while only very weak binding was observed for 22H8^LH-scFv-Fc^ (Fig. 4*B*). From these results, we decided to proceed with 13G5^HL-scFv-Fc^ and 22H8^HL-scFv-Fc^ due to their higher affinities for PTPRS ECD compared to their alternatively configured counterparts. We confirmed strong binding of both scFv-Fcs to the Fn9 fragment of PTPRS (Fig. 4*C*), with affinities comparable to those of the corresponding Abs (Fig. 4*D*). Both scFv-Fcs significantly inhibited migration of mouse FLS when used at 200 nM (Fig. 4*E*) and reduced PTPRS proximity to SDC4 and other PTPRS molecules as assessed by PLA (Fig. 4, *F* and *G*).

### Soluble PTPRS Fn9 domain acts as a decoy to displace PTPRS/SDC4 binding

**Figure 5:**
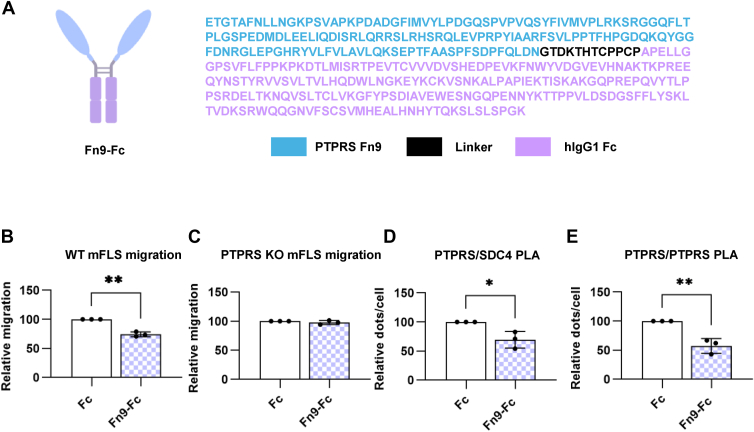
**hPTPRS Fn9-Fc fusions reduce FLS migration, PTPRS/SDC4 proximity and PTPRS clustering.***A*, schematic representation (*left*) and sequence (*right*) of the human PTPTRS Fn9-Fc fusion protein. *B* and *C*, relative transwell migration of WT (*B*) or *Ptprs* KO (*C*) mouse FLS in the presence of Fn9-Fc (1 μM). Each dot represents a biological replicate (n = 3). *D* and *E*, relative hPTPRS/mSDC4 co-localization (*D*) and hPTPRS/mPTPRS clustering (*E*) by proximity ligation assay (PLA) in the presence of Fn9-Fc (1 μM). Each dot represents a biological replicate (n = 3). Mean ± SEM are shown. ∗∗∗*p* ≤ 0.001, ∗∗*p* ≤ 0.01, and ∗*p* ≤ 0.05 by ordinary one-way ANOVA.

Because 13G5 binds an epitope on the PTPRS Fn9 domain, and the same domain has been proposed to contribute to the ECD's affinity to GAGs (28), we asked whether a soluble version of the isolated PTPRS Fn9 fused to human IgG1-Fc (Fn9-Fc) (Fig. 5*A*) could act as a decoy by displacing GAGs from cellular PTPRS, similar to Ig1&2. We tested this construct in migration assays with WT or *Ptprs* KO mFLS and showed that Fn9-Fc inhibited migration of mFLS in a PTPRS-dependent manner (Fig. 5, *B* and *C*). We then confirmed that Fn9-Fc acts *via* an analogous mechanism to Ig1&2 and the 13G5 Abs in both PTPRS/SDC4 and PTPRS/PTPRS PLA, as Fn9-Fc reduced the PLA signal in both cases (Fig. 5, *D* and *E*). These data suggest that Fn9-Fc can indeed enhance PTPRS cellular activity by interfering with its binding to SDC4 and subsequent PTPRS oligomerization.

### 13G5^IgG1^ decreases arthritis severity and improves histological parameters *in vivo*

**Figure 6:**
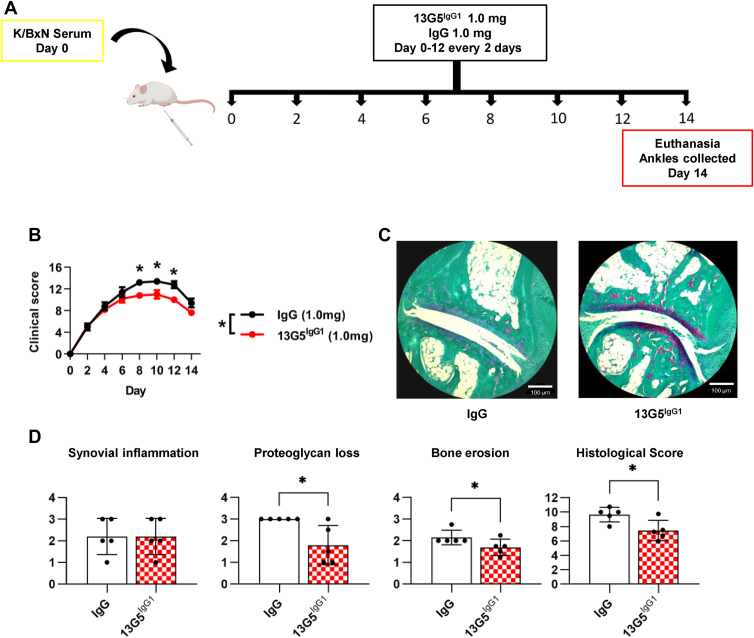
**13G5^IgG1^ decreases arthritis severity in mice.***A*, scheme representing the K/BxN STIA model and the dosing schedule. *B*, clinical score of STIA mice treated with 1.0 mg of mIgG1 control or 13G5^IgG1^; (n = 5). *C*, representative images of safranin-O staining. Scale bar 100 μm. *D*, histopathological evaluation of synovial inflammation, proteoglycan loss and bone erosion of hind ankles from mice in (*B*). Bone erosion, proteoglycan loss, and synovial inflammation were assessed separately, while histological score represents the sum of all parameters (n = 5). Mean ± SEM are shown. ∗*p* ≤ 0.05 by two-way ANOVA (*B*) and Mann–Whitney (*D*).

In previous studies, we demonstrated that soluble PTPRS Ig1&2 reduced arthritis severity in multiple mouse models (17, 21). Thus, we examined whether 13G5^IgG1^ would have a similar effect on inflammatory arthritis in mice using the K/BxN serum transfer-induced arthritis (STIA) model (31). Treatment with 13G5^IgG1^ (Fig. 6*A*) resulted in a statistically significant decrease in clinical score overall and on days 8, 10, and 12 (Fig. 6*B*). We then examined the histological parameters in the joints of mice subjected to the 13G5^IgG1^ treatment, finding that 13G5^IgG1^ significantly reduced proteoglycan loss, bone erosion, and, consequently, total histological score in mice when compared with IgG control (Fig. 6, *C* and *D*). Thus, despite the limited effect often observed with these first-generation Abs, our findings suggest that targeting the PTPRS-Fn9 region could be a viable strategy to mitigate RA disease progression.

## Discussion

RA is a common systemic autoimmune disease, causing joint inflammation, cartilage and bone damage, joint deformity, and disability (14). Multiple disease-modifying anti-rheumatic drugs are available for treating RA, and they all operate through suppression of immune responses (32, 33, 34). Targeting FLS in the joint is therefore being explored as an alternative or complementary approach to overcome some of the limitations of immunosuppressive therapy (16, 35). Over several years, our laboratory has been studying the molecular mechanisms behind FLS invasiveness with a particular focus on PTPRS inactivation and its role in promoting an aggressive and pro-inflammatory phenotype. Binding of the PTPRS (and other LAR-family RPTPs) ECD to HSPGs *via* their N-terminal membrane distal region induces clustering of the ECD in a mechanism termed the PG switch. Although direct evidence of its effect on intracellular catalytic activity is difficult to obtain, the PG switch is thought to modulate cellular functions by promoting intracellular clustering of the PTP catalytic domain and the subsequent loss of phosphatase activity (20). The structural basis for this effect however remains unclear. The highly flexible ECD of human PTPRS and other LAR-family RPTPs is made of up to 12 beads-on-a-string domains that can stretch over 40 nm when fully extended. It is thus reasonable to hypothesize the involvement of other ECD regions in the PG switch, as simple oligomerization of the N-terminal Ig1&2 moiety alone upon ligand binding may not be sufficient to drive formation of an intracellular inactive complex. Indeed, a report from the Geller group showed that while binding of GAGs to Ig1&2 is sufficient for ECD clustering, dysregulation of catalytic activity is dependent on a second binding site in the Fn9 domain even when an isoform that carries a shorter ECD is used (28). In its simplest form, a structural model for the PG switch would therefore entail recruitment of PTPRS to GAG-bearing SDC4, causing simultaneous binding and clustering of Fn9 and inactivation of the intracellular PTP domains. In this work, we show that 13G5, an Ab against an epitope in the PTPRS-Fn9 domain, is effective in reversing a pathogenic FLS phenotype. The effect of 13G5 was PTPRS- and SDC4-dependent, suggesting the PTPRS-Fn9 domain plays an active role in the PG switch. Indeed, in assays that tested PTPRS proximity to SDC4 or other PTPRS molecules, 13G5 disrupted PTPRS oligomerization and interaction with SDC4. A recombinant Fc fusion of the Fn9 domain similarly inhibited PTPRS proximity to other PTPRS molecules and SDC4 and FLS migration. These data show the potential for the Fn9 domain of PTPRS to be exploited for reversing the pathogenic behavior of FLS in RA and suggest multiple ways to approach PTPRS as a target for development of new RA therapeutics.

We found that of three Abs sharing the same epitope on Fn9, only 13G5 had a large effect on migration of FLS despite having the lowest *in vitro* affinity for the PTPRS ECD. On the other hand, scFv versions of 13G5 and 22H8 were similarly effective. Furthermore, our results suggest the constant regions of the Abs have a substantial effect on binding kinetics and antigen affinity, and indeed it is known that the isotype can, in some cases, influence the binding of Abs to their targets and even alter their specificity, although the mechanism by which this occurs is the subject of ongoing debate (36, 37). These observations point to a few options that can be explored, alone or in combination, to obtain Abs with improved biological effects: constant regions from different isoforms and species can replace the ones described; scFvs are amenable to *in vitro* maturation techniques and the design of bifunctional Abs to improve potency and specificity; knowledge of the epitope can facilitate *de novo* selection of new Abs by phage display; structure determination of Ab-antigen complexes can suggest ways to develop better species.

While we were able to provide strong evidence for binding of the Abs used in this study to PTPRS-Fn9, a structure-based rationale for the observed effects remains elusive. According to available structural predictions, the Fn9 epitope we identified and the experimentally determined Fn9 GAG binding site are situated on opposite sides of the Fn9 domain, which makes it unlikely that the effect of the Ab is mediated by direct competition with PG for PTPRS binding. Furthermore, interactions between the membrane distal, N-terminal domains of PTPRS with SDC4 occur at an even greater distance from this membrane proximal epitope, making direct competition even more unlikely in this case. Very little is known about the molecular and structural mechanism behind R2A PTP ECD oligomerization and PTP inactivation upon ligand engagement. Coles *et al.* showed the minimal Ig1&2 GAG binding unit to be dimeric *in vitro* (18); however, issues such as the involvement of direct inter- or intramolecular interactions between PTPRS domains, the polypeptide chain of SDC4 or third binding partners have not, to our knowledge, been investigated. To hint at a few possibilities, the Abs could act by disrupting a protein-protein interaction necessary for high affinity binding to HSPG; force an unfavorable spatial layout of GAG binding units; induce a conformational change in Fn9; constrain the intrinsic flexibility of the ECD. In the future, improved understanding of the impact of ECD structure and its interaction with ligands and targeting Abs on PTPRS activity will be key to developing new strategies to modify the activity of R2A PTPs for the study and management of RA and other pathologies affected by this class of enzymes.

## Materials and methods

### Antibodies and reagents

Rabbit anti-HA mAb (H6908) and mouse anti-Flag mAb (F1804) were purchased from Millipore Sigma. HRP-conjugated mouse anti-HA mAb (26183-HRP) was purchased from Invitrogen. Linear polyethyleneimine (PEI; PRIME) was purchased from Serochem. Mouse IgG1 (02-6100) and IgG2b (02-6300) were purchased from Invitrogen. Production of 9H5, 13G5, 22H8, 49F2, 13G5^IgG1^, and 22H8^IgG2b^ was contracted to Viva Biotech; VH and VL sequences from patent US10,730,955B2 were fused to the constant regions of mouse IgG1/κ or IgG2b/κ as indicated in Figures 1*B* and 3*A*.

### Mice

All animal experiments were carried out according to the Institutional Animal Care and Use Committee-approved protocol at UCSD (S16098). BALB/c (BALB/cAnNTac) and NOD (NOD/MrkTac) mice were acquired from Taconic Biosciences. *Ptprs* KO mice on BALB/c background and *Sdc4* KO mice on C57BL/6 background were generated as previously described (38).

### Cell culture and transfection

HEK293T cells were maintained in Dulbecco's modified eagle media (DMEM, Corning 10-013) supplemented with 10% fetal bovine serum (FBS). For transfection of HEK293T, 25 μg of DNA were pre-mixed with 37.5 μg PEI for 10 min in 2.5 ml DMEM and added to the cells with 17.5 ml DMEM supplemented with 2% FBS. Mouse fibroblast-like synoviocytes (mFLS) were isolated (17) from ankle joints of female 8-week-old WT, *Ptprs* KO BALB/C, or *Sdc4* KO C57BL/6 mice. mFLS were cultured in DMEM (Corning 10-017) with 10% FBS, 2 mM L-glutamine, and gentamicin (50 μg/ml; Life Technologies). Human FLS from patients with RA (RA FLS) were obtained from the UCSD Clinical and Translational Research Institute Biorepository. Each line was previously obtained from discarded synovial tissue from patients with RA undergoing synovectomy, as approved by the UCSD IRB under protocol no. 140175. The diagnosis of RA conformed to the ACR 1987 revised criteria (39). mFLS and RA FLS were used between passages 3 and 10 for all experiments and subjected to overnight starvation in 0.1% FBS (serum starvation medium) before functional assays. All media used contained 1% penicillin/streptomycin, 10 mM HEPES buffer, pH 7.0. All cultures were incubated at 37 °C in a humidified 5% CO_2_ atmosphere.

### Plasmids

Cloning and mutagenesis of full-length PTPRS was contracted out to GenScript. The open reading frame (ORF) encoding WT Human PTPRS (hPTPRS) isoform 1 (seq. ID Q13332, all hPTPRS numbering refers to this isoform) was cloned into pcDNA3.1 (+) between the AflII and XhoI restriction sites in-frame with a C-terminal HA tag. The other hPTPRS constructs were obtained from this by site-directed mutagenesis. WT mSDC4 (seq. ID O35988) was cloned into pcDNA3.1 (+) between the AflII and the XhoI restriction sites in-frame with a C-terminal Flag tag. WT mPTPRS (B0V2N1) was cloned into pcDNA3.1 (+) between the AflII and XhoI restriction sites in-frame with a C-terminal Flag tag. mPTPRS ectodomain plasmid was obtained by amplifying and subcloning the extracellular domain into pHLsec (AgeI, KpnI) using the forward/reverse primer pair CCACCGGTGAAGAACCACCCAGGTTTATCAGAG/CCGGGTACCCTCCTCGCCGTCCACAAT. mPTPRS Fn9 mutant was obtained from Genscript using the mPTPRS ectodomain plasmid as a template. ORFs encoding for all scFvs flanked by AgeI and KpnI restriction sites were synthesized by Genscript and subcloned into pHLsec as C-terminal hFc or 6xHis fusions. For hPTPRS Fn9 (residues 1117-1270) the corresponding DNA was amplified from WT hPTPRS with the forward/reverse primer pair GCGACCGGTACTGCCTTCAACCTGCTCAACG/GCCGGTACCGTTATCCAGCTGGAAGGGG and subcloned into pHLsec.

### Protein expression and purification

For in-house expression, HEK293T cells were transiently transfected with the appropriate plasmid and cultured in DMEM supplemented with 2% FBS or OptiMEM (Invitrogen 31985070) media. Supernatants were collected and replaced every 2 to 3 days for 7 to 10 days and stored at 4 °C. After the final collection, 6xHis-tagged WT and mPTPRS FN9His ECD was purified by Ni-NTA affinity followed by HiTrap heparin affinity chromatography (Cytiva 17524802) with a NaCl gradient elution using an NGC liquid chromatography system (Bio-Rad). 6xHis-tagged PTPRS Fn9 was purified by a single step Nickel affinity chromatography, followed by dialysis in 20 mM Tris, 150 mM NaCl, pH 8.0. All other Fc fusions were purified by Protein A affinity chromatography, eluted with IgG Elution buffer (ThermoFisher 21004) and immediately neutralized with 1:20 v/v 1 M Tris buffer, pH 9.5. Proteins were then concentrated to 1 to 20 mg/ml and their purity was verified by SDS-PAGE.

### Bio-layer interferometry (BLI)

A 4-channel GatorPilot (Gator Bio) system was used for BLI assays of binding kinetics and epitope binning. All assays were performed in Gator K buffer (0.02% BSA, 0.002% Tween-20 in PBS, pH 7.4) at 37 °C with shaking at 1000 rpm. For Abs binding kinetics to mPTPRS ECD and hPTPRS Fn9, the ligand was immobilized on anti-6xHis probes at 100 to 200 nM for 30 to 200 s and tips were washed for 120 s. Abs were diluted at concentrations of 10, 30, 50, 100, 200, 300, 400, and 500 nM (13G5 and 22H8) or 1, 5, 10, 20, 40, 60, 80, and 100 (49F2), and the signal was recorded for 200 to 400 s, followed by dissociation in K buffer. For anti-PTPRS scFv-Fc fusions binding to hPTPRS Fn9, concentrations of 1, 2.5, 5, 10, 20, 30, 40, 50 nM were used for 900 s. The 0 nM analyte condition was subtracted from each curve to correct for drift. A reference run without any loading was performed and subtracted from the main assay. Curve fitting was performed in Prism (GraphPad Software, Inc) using a biphasic (2:1) association model. Epitope binning assay was performed by loading mPTPRS ECD to the anti-His probes at 70 nM and subjecting probes to association 1 and association 2. The association 1 step was performed for 1200 s at 100 nM to ensure epitope near-saturation. A common epitope was assumed when no detectable association 2 was measured after association 1.

### Transwell migration assay

Confluent mFLS were starved for 24 h and harvested by light trypsin digestion and seeded at 50,000 cells in 100 μl serum-free DMEM containing 0.5% BSA in the upper chamber of a 6.5-mm-diameter Transwell polycarbonate culture insert with a pore size of 8 μm (Costar 3422). Inserts were placed in 24-well plates with 600 μl DMEM supplemented with 10% FBS. The plates were incubated for 24 h at 37 °C, after which the transwell inserts were removed and the upper chamber was gently wiped with a cotton swab to remove non-migrated cells. Transwell membranes were fixed for 5 min in pre-chilled (−20 °C) methanol, stained in 0.5% crystal violet in 25% methanol for 30 min, rinsed and imaged using a Motic AE200 inverted microscope at 10× magnification. Cell migration was quantified by counting stained cells in four random non-overlapping fields in three wells per biological replicate.

### Proximity ligation assay

Proximity ligation assay (PLA) was performed using the Duolink *in situ* Detection Reagents Red kit (#DUO92007, Sigma) according to the Duolink PLA Fluorescence Protocol. Cells were fixed with 4% paraformaldehyde for 15 min, permeabilized with 0.1% Triton X-100 at room temperature for 10 min, blocked in a drop of Blocking Solution (Sigma-Aldrich DUO82007) at 37 °C for 1 h and incubated with primary Ab (rabbit anti-HA [Sigma-Aldrich, H6908] or mouse anti-Flag [Sigma-Aldrich, F1804]) diluted in Duolink Ab Diluent (Sigma-Aldrich DUO82008) for 1.5 h at 37 °C. After washing in wash buffer A cells were incubated with secondary Ab conjugated with PLUS or MINUS probes (Sigma-Aldrich) for 1 h at 37 °C. Anti-Mouse PLUS (#DUO92001) and Anti-Rabbit MINUS (#DUO92005) were used as secondary Abs. Cells were then washed twice in wash buffer A and incubated with the ligase (1:40 in ligation buffer) for 30 min at 37 °C, washed again and incubated with polymerase (1:80 in an amplification buffer) for 100 min at 37 °C. Finally, cells were washed in wash buffer B (200 mM Tris, mM NaCl), mounted in DuoLink *in situ* mounting medium with DAPI (Sigma-Aldrich, DUO82040), and allowed to dry before imaging. Negative controls were performed using secondary Abs only. For PTPRS/SDC4 PLA, HEK293T cells were co-transfected with HA-tagged human PTPRS (WT or mutant), and Flag-tagged mouse SDC4. For dimerization PLA, HEK293T cells were co-transfected with HA-tagged human PTPRS (WT or mutant), and Flag-tagged mouse PTPRS. In both PLAs, cells were detached 1 day after transfection by pipetting and plated on 15 mm round coverslips, then allowed to adhere overnight and treated with Abs (anti-PTPRS or IgG) at 200 nM or Ig1&2 or Fc control at 20 nM for 30 min before the PLA protocol.

### Hydrogen-deuterium exchange mass spectrometry (HDX-MS)

Purified mPTPRS ECD was first used to optimize the quenching conditions for HDX experiments using an immobilized pepsin column (16 μl bed volume). The coverage maps of identified peptides were compared, and 0.8 M GuHCl/80 mM tris(2-carboxyethyl)phosphine quench buffer was selected for the exchange experiments. Coverage of the polypeptide sequence was 100%. All exchange stock solutions contained 1.2 mg/ml protein, 8.3 mM Tris, 150 mM NaCl, pH 7.2, and kept on ice. Exchange experiments were initiated by adding 66 μl exchange stock solutions to 198 μl D_2_O-containing buffer (8.3 mM Tris, 150 mM NaCl, pDread 7.2) and incubating for various times (10, 100, 1000, 10,000 s) at 0 °C. At each indicated time, 16 μl exchange reaction solution was taken out, mixed with 24 μl quench buffer, and frozen on dry ice after incubating on ice for 5 min. Non-deuterated and equilibrium deuterated control samples were also prepared for back exchange correction. All frozen samples were thawed at 4 °C and subjected to proteolysis and LC/MS analysis. All the columns were kept at 0 °C to minimize back exchange. The deuterium incorporation of deuterated peptides was determined using HDXaminer (Sierra Analytics, LLC), which calculates the centroid values of each peptide. Back-exchange corrections were calculated. Ribbon Maps were generated with an in-house Excel macro and MatLab scripts.

### Immunoprecipitation, SDS-PAGE, and Western blot

HEK293T cells overexpressing WT full length or truncated hPTPRS (PTPRS^Δ^^32^^6^, PTPRS^Δ^^109^^6^, PTPRS^Δ^^11^^60^) were washed twice with ice-cold PBS and lysed with radioimmunoprecipitation buffer (RIPA) (10 mM Tris-HCl, 1 mM EDTA, 0.5 mM EGTA, 1% Triton X-100, 0.1% sodium deoxycholate, 0.1% SDS, 140 mM NaCl, pH 8.0), supplemented with Pierce proteinase inhibitor (Thermo Fischer A32963). Lysates were centrifuged for 30 min at 4 °C at 21,000 rcf and the supernatants were collected and incubated with Abs for 2 h at 4 °C. Protein G Sepharose beads were added, the suspension was incubated for 2 h at 4 °C with gentle mixing, the beads were washed three times with cold PBS and eluted with 2× Laemmli buffer containing 10% β-mercaptoethanol followed by SDS-PAGE in a 4 to 20% 1.0 mm Tris-Glycine gel (Invitrogen, XP04205BOX) and transferred to a nitrocellulose membrane. Membranes were blocked with 5% BSA for 1 h at room temperature and probed with HRP-conjugated anti-HA Ab (Thermo Fisher, 26183-HRP). Protein bands were visualized by chemiluminescence on a Syngene GeneGnome XRQ imager. Signal intensity was quantified using ImageJ software.

### Surface plasmon resonance

Surface plasmon resonance binding studies were carried out on an Octet SF3 (Sartorius) instrument. The anti-PTPRS Ab was immobilized on Fc1 of a PCH Sensor Chip (Sartorius) by amine coupling. The Fc2 channel was used as the reference. The assay buffer includes buffer 1× PBS supplemented with 0.005% Tween-20. A series of concentrations of the analyte mPTPRS ECD, ranging from 2500 nM to 9.77 nM, were prepared in the assay buffer. Binding kinetics were measured following a multi-cycle kinetics protocol. Regeneration was performed with 10 mM glycine-HCl, pH 2.5. The injection parameters for all the analyte and buffer cycles were 50 μl/min flow rate, 120 s association time, 300 s dissociation time. Data were processed with the Octet SPR analysis software.

### Flow-cytometry binding assay

HEK293T cells were transfected with hPTPRS-HA or empty vector (pcDNA3.1-HA) for 48 h. 500,000 cells were transferred to a 96-well plate and incubated for 1 h with 13G5 and 22H8 at the indicated concentrations. Cells were then washed three times with FACS buffer and stained with AF568-labelled anti-mouse IgG Ab (Invitrogen, A-11004) for 30 min. For intracellular staining, cells were washed three times and fixed with IC fixation buffer (Invitrogen, 00-8222-49) and permeabilized with a permeabilization buffer (Invitrogen, 00-8333-56). HA-tag staining was performed by incubating cells with the AF488-labelled anti-HA Ab (Cell Signaling, 2350) for 1 h. Cells were then washed three times and resuspended in FACS buffer for analysis in a Sony ID7000 Spectrum Cell Analyzer. Data were analyzed using FlowJo.

### Enzyme-linked immunosorbent assay (ELISA)

2.5 μg/ml mPTPRS ECD in 50 mM bicarbonate buffer pH 9.5 was used to coat the wells of a 384-well clear bottom plate at 4 °C overnight. Wells were blocked with 1× bovine serum albumin blocking buffer for 1 h at room temperature and incubated with the indicated concentrations of the scFv-hFc fusion proteins. The wells were then washed and incubated with 1:20,000 HRP-conjugated anti-human IgG1 (R&D Systems, HAF109) for 1 h at room temperature. After washing, tetramethylbenzidine solution (R&D Systems, DY999B) was added to the wells and the absorbance at 450 nm was measured after the appropriate time using a Tecan Spark plate reader.

### Serum transfer-induced arthritis (STIA) model

C57BL/6 KRN mice provided by Dr Christophe Benoist (Harvard Medical School) and NOD mice were crossed to obtain offspring that developed arthritis at around 6 to 7 weeks of age (spontaneous K/BxN mice). Serum from arthritic K/BxN mice was pooled for use in the K/BxN STIA model (31). Eight- to 12-week-old mice were injected retro-orbitally with 150 μl K/BxN serum on day 0. Arthritis clinical score was measured as previously described (21), scoring 0 = normal; 1 = minimal erythema and mild swelling; 2 = moderate erythema and mild swelling; 3 = marked erythema and severe swelling, digits not yet involved; 4 = maximal erythema and swelling, digits involved. Measurement of ankle thickness was performed every other day. Antibody or isotype control administration was performed as described in Figure Legends.

### Histological score of mouse arthritic joints

Whole hind paws were fixed in 10% formalin, decalcified, trimmed, and embedded. Sections were prepared from tissue blocks and stained with H&E and Safranin-O according to the “SMASH” recommendations (40). Histopathological scoring was performed as described (40). Briefly, joints of arthritic mice were assigned scores of 0 to 3 for inflammation based on H&E staining, according to the following criteria: 0 = normal; 1 = minimal infiltration of inflammatory cells in the periarticular area; 2 = mild infiltration; 3 = marked infiltration. Joints of arthritic mice were given scores of 0 to 3 for bone erosion based on H&E staining, according to the following criteria 0 = healthy; 1 = Small bone erosion (superficial bone erosion at the outer surface of the cortical bone); 2 = Enhanced bone erosion (subchondral bone erosions, partial or complete penetration of cortical bone, small breakthrough of cortical bone to bone marrow); 3 = Massive bone erosion (extended synovial pannus invasion mostly causing complete breakthrough of the cortical bone to the bone marrow cavity, loss of bone architecture). Proteoglycan loss was identified by diminished Safranin O staining of the matrix and was scored on a scale of 0 to 3, where 0 = Healthy (intact cartilage consisting of fully Safranin-O); 1 = Mild (loss of staining in approximately one-third of the superficial cartilage zone, superficial cartilage layer is still predominantly reddish); 2 = Moderate (loss of red staining in up to two-thirds of the superficial cartilage zone, destaining predominates throughout the remaining stained areas). Histological analyses were performed in a blind manner. Images of whole ankles were acquired using the ECHO Revolve Microscope (ECHO) and analyzed by ImageJ software.

### Statistical analysis

Statistical analyses were performed in GraphPad Prism 9.0.2. Data were normalized to the respective control and ordinary one-way or two-way ANOVA was used to assess the significance of variables that were found to be normally distributed based on the Shapiro–Wilk test. The Kruskal–Wallis or Mann–Whitney *U* test was used for the nonparametric statistics. Data were considered statistically significant when *p* < 0.05.

## Data availability

All data are contained within the manuscript or will be made available upon written request to the corresponding authors.

## Supporting information

This article contains supporting information.

## Conflict of interest

The authors declare the following financial interests/personal relationships which may be considered as potential competing interests: NB holds equity in Knoubis Bio, Inc. SMS holds equity in and receives income from Knoubis Bio, Inc.
