# Supplementary material for: Modulation of the PTPRS proteoglycan switch by antibodies binding to the membrane-proximal fibronectin-type III domain
Source: J Biol Chem. 2025 Jul 10;301(8):110470. doi: 10.1016/j.jbc.2025.110470 (PMC12363564; doi:10.1016/j.jbc.2025.110470)
Supplement: Supporting information [file mmc1.pdf]

A

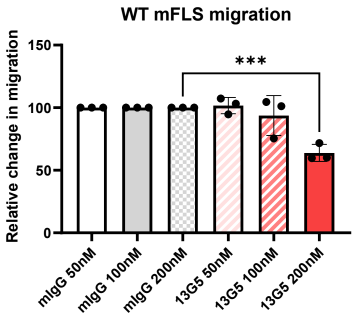


B

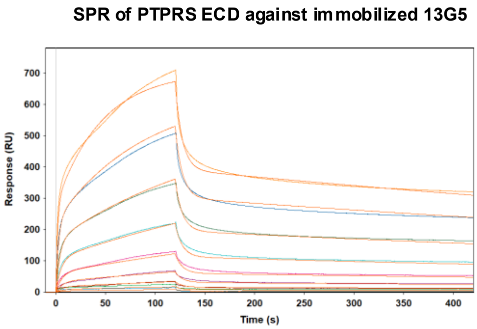


| $K_D$ (nM) | $K_{on}$ (1/M*s) | $K_{off}$ (1/s) |
|---|---|---|
| 94.6±0.5 | $8.35 \pm 0.01 \times 10^{3}$ | $7.9 \pm 0.04 \times 10^{-4}$ |

**Figure S1. 13G5 dose-response migration and surface plasmon resonance (SPR) for the PTPRS ECD against immobilized 13G5. (A)** Relative transwell migration of mouse FLS in the presence of 13G5 at the indicated concentrations. Mouse IgG are negative controls. Each dot represents a biological replicate (n=3). **(B)** SPR response curves and the fit for the multi-cycle binding kinetics data. Response curves colored by the analyte concentration: orange, blue, green, cyan, pink, light purple, brown, light green represent Ectodomain concentrations of 2500, 1250, 625, 312, 156, 78, 39.1, 19.5, and 9.77 nM, respectively. Fitted curves are in orange. Mean ± SEM are shown. ***P ≤ 0.001 by ordinary one-way ANOVA (A).

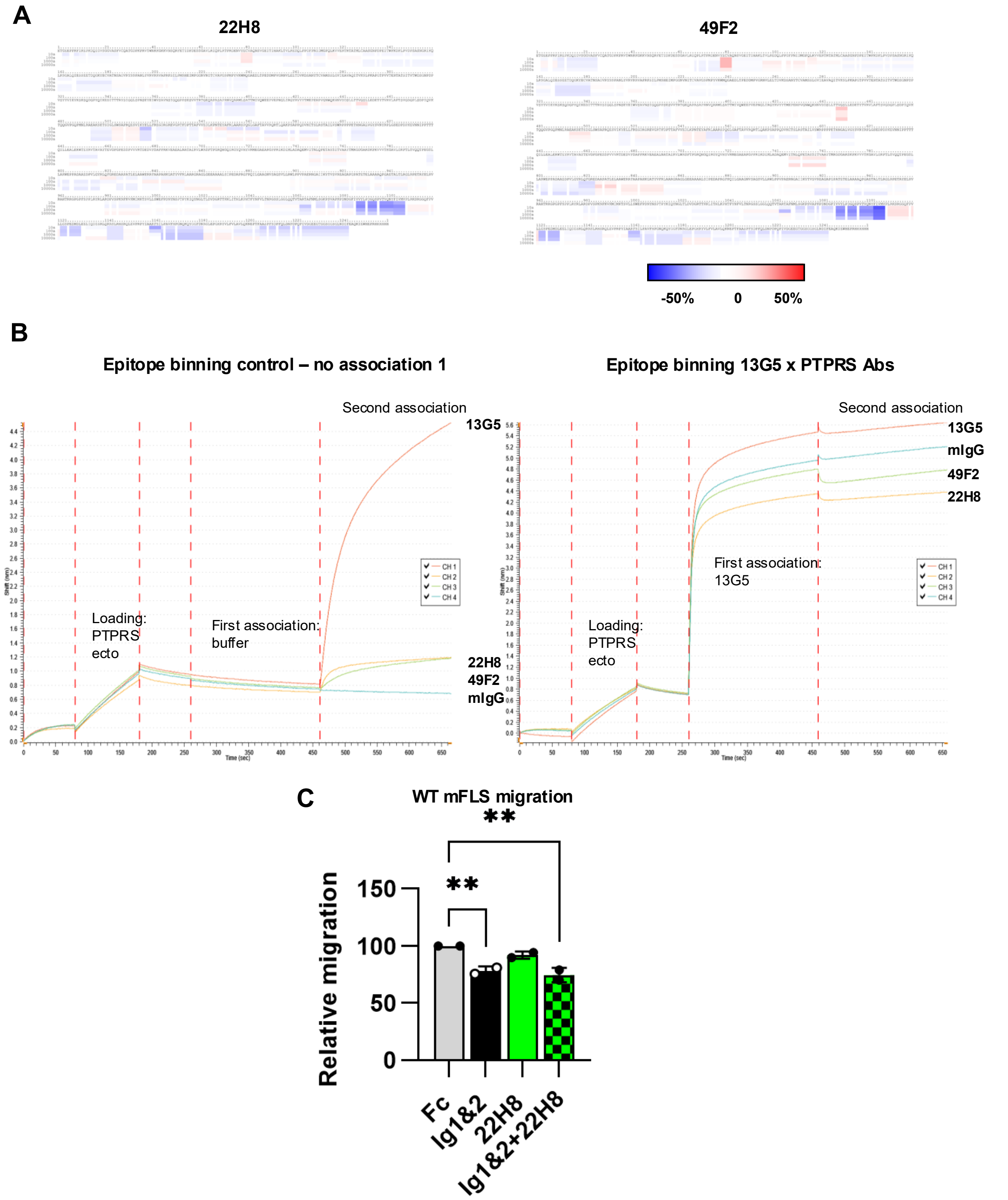


**Figure S2. Anti-PTPRS HDX-MS and Epitope binning: (A)** Hydrogen-Deuterium exchange mass spectrometry (HDX-MS) of mouse PTPRS ectodomain in the presence or absence of 22H8 (left), and 49F2 (right). Ribbon diagrams show the difference between PTPRS alone and PTPRS + Ab (top, blue indicates peptides that exchange faster in the absence of the Ab) **(B)** Epitope binning of 13G5, 22H8, and 49F2 measured by biolayer interferometry. 13G5, 22H8, 49F2 or control IgG1 were bound to immobilized mPTPRS ectodomain after the first association under near-saturating conditions with 13G5 (right). Control run without first association was performed in order to compare Abs shift (left). Data are representative of three independent experiments. **(C). )** Relative transwell migration of mouse FLS in the presence of 22H8, Ig1&2, or both 22H8 and Ig1&2 (22H8: 200 nM; Ig1&2: 20 nM. **P ≤ 0.01 by ordinary one-way ANOVA

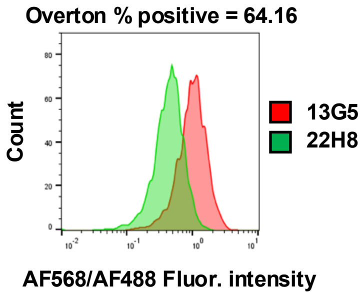


**Figure S3. Binding of 13G5 and 22H8 to HEK293 cell surface.** Representative flow cytometry histogram showing 13G5 or 22H8 binding to WT PTPRS overexpressed on HEK293T cells. The Overton percentage was calculated by subtracting the control histogram. AF568/AF488 represents the ratio of antibody to overexpressed PTPRS.
